# Supplementary material for: Impact of Adjuvant Modalities on Survival in Patients with Advanced Stage Endometrial Carcinoma: A Retrospective Analysis from a Tertiary Medical Center
Source: Int J Environ Res Public Health. 2019 Jul 18;16(14):2561. doi: 10.3390/ijerph16142561 (PMC6678420; doi:10.3390/ijerph16142561)
Supplement: Supplementary file 1 [file ijerph-16-02561-s001.pdf]

International Journal of
Environmental Research and Public Health

MDPI

Article - supplementary material

Impact of Adjuvant Modalities on Survival in Patients with Advanced Stage Endometrial Carcinoma: A Retrospective Analysis from a Tertiary Medical Center

Yi-Jou Tai [1,2], Heng-Cheng Hsu [1,2,3], Ying-Cheng Chiang [1,2], Yu-Li Chen [1,4], Chi-An Chen [1] and Wen-Fang Cheng [1,2,5,*]

1 Department of Obstetrics and Gynecology, College of Medicine, National Taiwan University, Taipei 100, Taiwan
2 Graduate Institute of Clinical Medicine, College of Medicine, National Taiwan University, Taipei 100, Taiwan
3 Department of Obstetrics and Gynecology, National Taiwan University Hospital Hsin-Chu Branch, Hsin-Chu City 300, Taiwan
4 Graduate Institute of Anatomy and Cell Biology, College of Medicine, National Taiwan University, Taipei 100, Taiwan
5 Graduate Institute of Oncology, College of Medicine, National Taiwan University, Taipei 100, Taiwan
* Correspondence: wenfangcheng@yahoo.com; Tel.: +886-2-2312-3456

Table S1. Univariate analysis for survival of grade 1/2 and grade 3 tumors in advanced-stage patients.

	**Grade 1/2**				**Grade 3**			
	PFS		OS		PFS		OS	
Variable	HR (95% CI)	*p*	HR (95% CI)	*p*	HR (95% CI)	*p*	HR (95% CI)	*p*
**Age ≥ 60 (years)**	1.03 (0.49,2.17)	0.93	0.75 (0.25,2.29)	0.61	1.68 (0.89,3.18)	0.11	1.66 (0.75,3.66)	0.21
**Menopause**								
No	1	0.40	1	0.97	1	0.14	1	0.53
Yes	1.36 (0.66-2.81)		1.02 (0.41,2.55)		1.64 (0.85,3.19)		1.30 (0.57,2.95)	
**FIGO stage**								
IIIA	1		1		1		1	
IIIB	3.54 (0.88,14.3)	0.08	3.66 (0.32,41.6)	0.30	0.72 (0.17,3.01)	0.65	0.44 (0.08,2.52)	0.36
IIIC (IIIC1+IIIC2)	1.54 (0.59,4.01)	0.38	4.48 (0.97,20.7)	0.06	0.88 (0.33,2.38)	0.80	0.52 (0.16,1.69)	0.28
IV (IVA+IVB)	5.43 (1.94,15.2)	<0.01	9.16 (1.67,50.1)	<0.01	1.69 (0.61,4.66)	0.31	1.25 (0.38,4.09)	0.71
**Myometrial invasion**								
<1/2	1	0.03	1	0.63	1	0.63	1	0.78
≥1/2	2.35 (1.09,5.05)		1.26 (0.50,3.13)		1.19 (0.59,2.37)		1.14 (0.48,2.71)	
**Presence of LVSI**	2.13 (0.99,4.59)	0.05	1.53 (0.60,3.94)	0.37	0.95 (0.37,2.43)	0.92	1.17 (0.35,3.92)	0.80
**Lymph node metastases**	1.71 (0.75,3.88)	0.20	3.74 (1.06,13.2)	0.04	0.89 (0.42,1.89)	0.76	0.98 (0.36,2.65)	0.97

**Residual tumor size**								
≥1 cm	1	<0.01	1	<0.01	1	0.37	1	0.32
<1 cm	0.19 (0.09,0.40)		0.23 (0.08,0.63)		0.74 (0.38,1.43)		0.67 (0.30,1.49)	
**Adjuvant therapy**								
Radiation or CCRT	1		1		1		1	
Chemotherapy	2.44 (1.13,5.29)	0.02	2.32 (0.89,6.10)	0.09	1.11 (0.58,2.11)	0.76	0.90 (0.39,2.08)	0.81
Combined modalities	3.01 (1.11,8.17)	0.03	0.74 (0.09,5.99)	0.78	0.45 (0.15,1.34)	0.15	0.48 (0.14,1.73)	0.26

PFS, progression-free survival; OS, overall survival.

© 2019 by the authors. Submitted for possible open access publication under the terms and conditions of the Creative Commons Attribution (CC BY) license (http://creativecommons.org/licenses/by/4.0/).
